# Risk factors for febrile neutropenia and effectiveness of primary prophylaxis with pegfilgrastim in patients with esophageal cancer treated with docetaxel, cisplatin, and 5-fluorouracil

**DOI:** 10.1186/s12957-019-1665-x

**Published:** 2019-07-17

**Authors:** Yu Ohkura, Masaki Ueno, Harushi Udagawa

**Affiliations:** Department of Gastroenterological Surgery, Toranomon Hospital and Okinaka Memorial Institute for Medical Research, 2-2-2 Toranomon, Minato-ku, Tokyo, 105-8470 Japan

**Keywords:** Pegfilgrastim, Esophageal cancer, DCF regimen, Febrile neutropenia

## Abstract

**Background:**

The docetaxel, 5-fluorouracil, and cisplatin (DCF) regimen is an effective form of chemotherapy for advanced esophageal cancer. However, the incidence of adverse events such as febrile neutropenia and hematological toxicity is high.

**Methods:**

Among 937 patients with esophageal cancer at Toranomon Hospital between January 2011 and December 2018, 92 who underwent the DCF regimen as initial treatment were selected. We investigated the risk factors for febrile neutropenia in patients with esophageal cancer treated with DCF regimen and the effectiveness of pegfilgrastim as primary prophylaxis.

**Results:**

Adverse events (CTCAE grade ≥ 3) were observed in 45 of the 92(48.9%) patients with esophageal cancer treated with an initial DCF regimen. Febrile neutropenia was observed in 20 (21.7%) patients. Non-use of pegfilgrastim (odds ratio = 16.393; 95% confidence interval 2.049–125.0) as primary prophylaxis was identified as an independent factor predictive of febrile neutropenia. The pegfilgrastim group had a significantly lower incidence of neutropenia than the control group (9.1% vs 61.0%; *p* < 0.001). The incidence of febrile neutropenia was 3.0% in the pegfilgrastim group and 32.2% in the control group (*p* = 0.001). In addition, the incidence of any adverse effect ≥ CTCAE grade3 was also significantly lower in the pegfilgrastim group (12.1% vs 69.5%; *p* < 0.001). The reduced/interruption rate of next DCF therapy was 6.1% in the pegfilgrastim group and 30.5% in the control group (*p* = 0.006).

**Conclusion:**

This study revealed that the non-use of pegfilgrastim was an independent factor predictive of febrile neutropenia in multivariate analysis. Pegfilgrastim as primary prophylaxis prevents severe neutropenia and febrile neutropenia in patients with esophageal cancer treated with the DCF regimen.

## Introduction

Esophageal cancer is one of the most malignant forms of gastrointestinal cancer. The efficacy of chemoradiotherapy and chemotherapy has been demonstrated, and so they are widely used to treat esophageal cancer. Reports have also documented the efficacy of preoperative chemotherapy, which has become the standard form of treatment in Japan. Currently, the standard treatment for stage II and III esophageal cancer is preoperative chemotherapy (cisplatin + 5-fluorouracil [5-FU]: CF regimen) + radical surgery. According to a previous report [1], the 5-year survival rate for cT1-2 cancer is favorable at 79% following preoperative treatment with the CF regimen. In contrast, the 5-year survival rate for cT3 is poor at 49%, with results showing poorer efficacy of the CF regimen in more advanced cases. A more potent form of preoperative treatment is therefore desirable. For this reason, there has been a growing number of reports on the DCF regimen (docetaxel + CF) as this is the more potent form of treatment in recent years [2–4], and the high success rate has attracted attention. On this background, we are currently in the late stages of JCOG1109, a phase 3 comparative clinical trial [5] to clarify the superiority of the preoperative DCF regimen and preoperative CF chemoradiotherapy compared to the standard preoperative CF regimen. The DCF regimen, which is more potent than the conventional CF regimen, is associated with high rates of febrile neutropenia, which is viewed as problematic. Although the efficacy of pegfilgrastim for preventing febrile neutropenia has been established in other fields such as hematological diseases, it has not yet been in patients undergoing the DCF regimen as a form of treatment for esophageal cancer. For this reason, in this study, we investigated the risk factors for febrile neutropenia in addition to performing an analysis of pegfilgrastim-treated cases at our hospital and investigated the effectiveness of the DCF regimen.

## Materials and methods

### Study population

This single-center retrospective study was conducted to evaluate the risk factors for febrile neutropenia in patients with esophageal cancer who were treated with the DCF regimen and the effectiveness of primary prophylaxis with pegfilgrastim. A total of 937 consecutive patients with esophageal cancer were identified from a database that was prospectively constructed between January 2011 and December 2018. Among these, 92 patients were selected according to the following eligibility criteria. Inclusion criteria were histopathologically proven squamous cell carcinoma, esophageal cancer from cervical esophagus to abdominal esophagus, and patients who underwent DCF regimen (100% dose) as initial treatment; exclusion criteria were any history of esophageal treatment or any history of chemotherapy for other cancer. All patients did not use prophylactic antibiotic therapy. Of these 92 patients, 20 patients who were diagnosed with febrile neutropenia as an adverse event were allocated to the febrile neutropenia (FN) group and the remaining 72 patients were allocated to the non-febrile neutropenia (N) group in study 1. Of these 92 patients, 33 patients were administered pegfilgrastim as primary prophylaxis and the remaining 59 patients were allocated to the control group in study 2. We investigated risk factors for febrile neutropenia in patients with esophageal cancer treated using the DCF regimen in study 1, and the effectiveness of pegfilgrastim as primary prophylaxis of adverse events and the advantage of pegfilgrastim use in DCF therapy in study 2. We graded all adverse events based on the Common Terminology Criteria for Adverse Events (CTCAE) ver. 4.03 [6], and grade ≥ 3 events were documented as targets for examination. This study was conducted with approval from the Institutional Review Board of Toranomon Hospital (approval number 1746).

### Pretreatment examination

Pretreatment examination involved physical examination, tumor marker testing, esophagography, upper gastrointestinal endoscopy, colonoscopy, endoscopic ultrasonography, computed tomography, positron emission tomography, and abdominal/neck ultrasonography. We performed gastrostomy before chemotherapy for patients who had dysphagia caused by a bulky esophageal tumor, especially T4 or suspicious T4 tumor. Disease stage was classified according to the UICC TNM grading system, 7th edition [7].

### Chemotherapy

All patients in this study received DCF therapy as initial treatment for esophageal cancer. The regimen in this study was docetaxel as 1-h 75 mg/m^2^ I.V. infusion on day 1, cisplatin 75 mg/m^2^ I.V. infusion on day 1 with standard I.V. hydration, and 5-FU 750 mg/m^2^/day by continuous infusion over 5 days. Standard antiemetic support including 5-HT3 antagonists, corticosteroids, and dopamine antagonists was provided. In some cases, pegfilgrastim (3.6 mg) 24 h post-completion of the 5-FU infusion for all cycles of DCF therapy was administered. The pegfilgrastim was adopted in our hospital in December 2014. Therefore, all these patients of pegfilgrastim group are cases from December 2014.

### Statistics

The risk factors for febrile neutropenia in patients with esophageal cancer treated by DCF therapy were assessed by bivariate logistic regression analysis. Comparisons between groups were performed using the Mann-Whitney *U* test and Pearson’s chi-squared test for statistical significance. Variables with significance of *p* < 0.10 in univariate analysis were entered into the bivariate logistic analysis. In bivariate analysis, *p* < 0.05 was considered significant. All analysis was performed using the Statistical Package for the Social Science (SPSS) software version 19.0J for Windows (SPSS Inc., Chicago, IL).

## Results

### Patient characteristics and univariate analysis of the 92 patients

Of the 92 patients, febrile neutropenia was observed in 20 (21.7%) patients. These 20 patients were allocated to the FN group, and the remaining 72 patients were allocated to the N group. Baseline characteristics of these two groups are summarized in Table 1. All 92 patients who underwent DCF therapy as initial treatment had a median age of 66.5 years, and 85.9% of patients were male. Median body mass index of this population was 21.1 kg/m^2^. Of 92 patients, 23 patients treated 1 cycle, 61 patients treated 2 cycles, 7 patients treated 3 cycles, and one patient treated 5 cycles. Univariate analysis between the two groups showed a significant difference in the use of pegfilgrastim (*p* = 0.001) and age (*p* = 0.032). Other patient characteristics, pretreatment hematological parameters, and tumor factors showed no significant difference between the two groups.

**Table 1:** Patient characteristics and results of univariate analysis of factors predicting febrile neutropenia

	Total (*n* = 92) *n*/median	FN group (n = 20)	N group (*n* = 72)	*p* value
Patients characteristics				
Age (years)	66.5	58.0	67.0	0.032
Sex (male/ female)	79/13	17/3	62/10	n.s.
PS (0/1/2)	71/19/2	16/3/1	55/16/1	n.s.
Alcohol (yes/no)	83/9	18/2	65/7	n.s.
Smoking (yes/no)	79/13	17/3	62/10	n.s.
Brinkman index (< 600/≥ 600)	40/52	6/14	34/38	n.s.
Malignancy	83/9	19/1	64/8	n.s.
Use of pegfilgrastim (yes/no)	33/59	1/19	32/40	0.001
Cardiovascular disease (yes/no)	6/86	1/19	5/67	n.s.
Pulmonary disease (yes/no)	8/84	3/17	5/67	n.s.
BMI	21.1	20.8	21.1	n.s.
Pretreatment hematological items				
WBC (× 10^3^/mL)	6.50	5.95	6.70	n.s.
Neutrophils (× 10^3^/mL)	4.22	3.66	4.68	n.s.
Hb (g/dL)	13.5	14.0	13.4	n.s.
Plt (× 10^3^/mL)	267.0	232.0	274.0	0.075
TP (g/dL)	7.1	7.2	7.1	n.s.
Alb (g/dL)	3.9	4.0	3.8	n.s.
BUN (mg/dL)	13.0	13.0	13.0	n.s.
Cre (mg/dL)	0.75	0.70	0.77	0.058
AST (IU/L)	20.0	21.5	19.0	n.s.
ALT (IU/L)	14.0	18.5	13.0	n.s.
LDH (IU/L)	181.0	173.5	181.0	n.s.
ALP (IU/L)	199.0	196.0	199.0	n.s.
γ s. (IU/L)	38.0	38.5	38.0	n.s.
Bil (mg/dL)	0.70	0.75	0.60	n.s.
CRP (mg/dL)	0.10	0.05	0.10	n.s.
Na (mmol/L)	142	142	142	n.s.
K (mmol/L)	4.2	4.3	4.2	n.s.
Cl (mmol/L)	105.0	105.0	105.0	n.s.
HbA1c (%)	5.6	5.6	5.6	n.s.
CEA (μg/L)	2.8	2.5	2.8	n.s.
CA19-9 (U/mL)	11.0	8.0	12.0	n.s.
SCC (μg/L)	1.3	1.2	1.3	n.s.
CYFRA (μg/L)	1.9	2.3	1.8	0.084
p53 U/mL	0.69	0.69	0.69	n.s.
Tumor factors				
c T factor (7th)				n.s.
1a/1b	2/7	0/0	2/7	
2	17	4	13	
3	50	14	36	
4a	5	2	3	
4b	11	0	11	
c N factor (7th)				n.s.
0	9	2	7	
1	33	6	27	
2	38	9	29	
3	10	3	7	
4	2	0	2	
c stage (7th)				n.s.
I (IB)	3	0	3	
II (IIA, IIB)	6/10	3/2	3/8	
III (IIIA, IIIB, IIIC)	21/13/16	5/2/2	16/11/14	
IV	23	6	17	
Tumor localization				n.s.
Ut	10	1	9	
Mt	50	12	38	
Lt	17	5	12	
Ae	2	0	2	
EGJ	13	2	11	

*Abbreviations*: *PS* performance status, *BMI* body mass index, *WBC* white blood cell count, *Hb* hemoglobin, *Plt* platelet count, *TP* total protein, *Alb* albumin, *BUN* blood urea nitrogen, *Cre* creatinine, *AST* aspartate transaminase, *ALT* alanine aminotransferase, *LDH* lactate dehydrogenase, *ALP* alkaline phosphatase, *GTP* glutamyl transpeptidase, *Bil* bilirubin, *CRP* c-reactive protein, *Na* sodium, *K* potassium, *Cl* chloride, *CEA* carcinoembryonic antigen, *CA19-9* carbohydrate antigen 19-9, *SCC* squamous cell carcinoma antigen, *CYFRA* cytokeratin-19 fragments, *Ut* upper thoracic esophagus, *Mt* middle thoracic esophagus, *Lt* lower thoracic esophagus, *Ae* abdominal esophagus, *EGJ* esophagogastric junction

### Study 1: Independent risk factors for febrile neutropenia in multivariate analysis

Variables with significance of *p* < 0.10 in univariate analysis models were entered into the bivariate logistic analysis. Multivariate analysis using the results of univariate analysis (Table 2) was performed for the following selected variables: age, sex, use of pegfilgrastim (yes/no), platelet count, creatinine levels, and cytokeratin 19 fragment (CYFRA). The non-use of pegfilgrastim (odds ratio [OR] = 16.393; 95% confidence interval [CI] 2.049–125.0; *p* = 0.008) was identified as an independent factor predictive of febrile neutropenia.

**Table 2:** Results of multivariate analysis of the factors predicting febrile neutropenia

Variables	Odds ratio	95% CI	*p* value
Non-use of pegfilgrastim	16.393	2.049–125.0	0.008

### Study 2: Adverse events after DCF therapy between the pegfilgrastim and control groups

Adverse events (CTCAE grade ≥ 3) were observed in 45 of the 92 (48.9%) patients with esophageal cancer treated with initial DCF therapy. The pegfilgrastim group (12.1%) had a significantly lower incidence of CTCAE grade ≥ 3 events than the control group (69.5%) (*p* < 0.001). The incidence of neutropenia was 9.1% in the pegfilgrastim group and 61.0% in the control group, respectively (*p* < 0.001). The incidence of febrile neutropenia was 3.0% in the pegfilgrastim group and 32.2% in the control group, respectively (*p* = 0.001). There was no significant difference in the incidence of hyponatremia, anorexia, and nausea between these two groups (Table 3). The rate of classical granulocyte-colony stimulating factor (G-CSF; filgrastim) use was 0% in the pegfilgrastim group and 52.5% in the control group, respectively (*p* < 0.001). The rate of reduced/ interruption of next cycle DCF therapy due to adverse effects was 6.1% in the pegfilgrastim group and 30.5% in the control group, respectively (*p* = 0.006). The reasons of reduced/ interruption of next cycle DCF therapy were 1 febrile neutropenia and 1 elevation of liver enzyme levels in the pegfilgrastim group, and 12 febrile neutropenia, 5 neutropenia, and 1 acute renal failure in the control group.

**Table 3:** Comparison of adverse events between the two groups

	Total (*n* = 92)	Pegfilgrastim (*n* = 33)	Control (*n* = 59)	*p* value
CTCAE ≥ grade 3				< 0.001
Yes	45	4	41	
No	47	29	18	
CTCAE grade 4				0.018
Yes	9	0	9	
No	83	33	50	
Neutropenia				< 0.001
Yes	39	3	36	
No	53	30	23	
Febrile neutropenia				0.001
Yes	20	1	19	
No	72	32	40	
Hyponatremia				0.643
Yes	4	1	3	
No	88	32	56	
Anorexia				0.216
Yes	7	1	6	
No	85	32	53	
Nausea				0.285
Yes	2	0	2	
No	90	33	57	
G-CSF (filgrastim) use				< 0.001
Yes	31	0	31	
No	61	33	28	
Next cycle DCF therapy				0.006
Planed administration	72	31	41	
Reduced/interruption	20	2	18	

## Discussion

In this study, we attempted to identify the factors predictive of febrile neutropenia in patients with esophageal cancer treated using initial DCF therapy. Study 1 shows that the non-use of pegfilgrastim was an independent factor predictive of febrile neutropenia in multivariate analysis. Although several background risk factors were suspected among the risk factors for febrile neutropenia in univariate analysis, only the absence of pegfilgrastim administration remained as an independent risk factor for febrile neutropenia through multivariate analysis. Although the FN group was significantly younger (*p* = 0.032) in univariate analysis, this can be explained by the fact that we performed DCF therapy for young patients at first, but came to perform DCF therapy for the elderly patients after the use of pegfilgrastim was started. In study 2, the pegfilgrastim group had a significantly lower incidence of both neutropenia and febrile neutropenia than the control group. Including these, the pegfilgrastim group had a significantly lower incidence of entire CTCAE grade ≥ 3 events. As a consequence, the pegfilgrastim group had less incidence of dose reduction in the following DCF therapy. Therefore, the use of pegfilgrastim can be an option for chemotherapy to achieve the goal of successful completion of scheduled chemotherapy with DCF regimen.

The results of long-term follow-up during the MRC-OE2 clinical trial, which allocated patients with resectable esophageal cancer into a neoadjuvant CF group and a surgery only group, showed that the hazard ratio for overall survival was 0.84 (*p* = 0.03) in a neoadjuvant CF group, which was significantly more favorable [8, 9]. Currently, based on the results of the Japan Clinical Oncology Group (JCOG) 9907 clinical trial [1], the standard treatment for stage II and III esophageal cancer is preoperative chemotherapy (cisplatin + 5-FU: CF regimen) + radical surgery. Furthermore, a meta-analysis compared studies of neoadjuvant chemotherapy to surgery alone reported that the hazard ratio for overall survival was 0.87 (*p* = 0.005) when all the preoperative chemotherapy groups were compared to the surgery alone group, suggesting the efficacy of preoperative chemotherapy [10]. Meanwhile, neoadjuvant chemoradiotherapy has been widely performed in Western countries; however, the survival of neoadjuvant chemotherapy followed by surgery in Japanese study appears not inferior to the western results of neoadjuvant CRT. The JCOG 9907 clinical trial subgroup analysis results [1] showed that the 5-year survival rate for cT1-2 cancer is favorable at 79% when treated with the CF regimen preoperatively. By contrast, the 5-year survival rate for cT3 is poor at 49%, with results showing poorer efficacy of the regimen in more advanced cases. A more potent form of preoperative treatment is therefore desirable. There have been increasing numbers of reports of using the DCF regimen (docetaxel + CF regimen) in practice as this more potent form of treatment in recent years [2–4], and the high success rate has attracted much attention. Against this background, we are currently in the advanced stages of a phase 3 comparative clinical trial JCOG1109 [5] to verify the superiority of the preoperative DCF regimen and preoperative CF chemoradiotherapy compared to the preoperative CF regimen, which is the standard form of treatment. The DCF regimen, which is more potent than the conventional CF regimen, causes high rates of febrile neutropenia, which is viewed as problematic. Febrile neutropenia is an adverse reaction to anticancer drug treatment that may have serious outcomes, and primary prophylaxis with pegfilgrastim is recommended when using regimens with a high rate of febrile neutropenia onset of ≥ 20%. Going forward, based on the results of the JCOG 1109 clinical trial [5], the DCF regimen may become a standard form of treatment, so it is needed before this occurs to rapidly establish an effective modality of prophylaxis for febrile neutropenia. For this reason, we performed an analysis of pegfilgrastim-treated cases at our hospital and investigated its effectiveness in the DCF regimen.

In September 2014, conventional G-CSF was pegylated to prolong its serum half-life, yielding pegfilgrastim; a single administration at a dose of 3.6 mg during each cycle is sufficient to prevent febrile neutropenia. Although the efficacy of pegfilgrastim for preventing febrile neutropenia has been demonstrated in other fields, such as hematological diseases, there are few reports assessing it in patients undergoing the DCF regimen as a form of treatment for esophageal cancer [2–4]. In this study, the administration of pegfilgrastim in practice resulted in a significantly lower incidence of adverse drug reactions ranked as CTCAE grade ≥ 3, and significantly fewer patients had neutropenia (*p* < 0.001) and febrile neutropenia (*p* = 0.001) in particular. No changes were observed in terms of other adverse events, such as hyponatremia, anorexia, and nausea. And also, we were able to decrease the rate of reduced/ interruption of next cycle DCF therapy due to adverse effects by using the pegfilgrastim from 30.5 to 6.1%, significantly (*p* = 0.006). There were no patients who administered additional classical G-CSF in the pegfilgrastim group; meanwhile, these occurred in 31 out of 59 subjects (52.5%).

In this study, of 92 patients, 77 patients underwent esophagectomy after neoadjuvant DCF therapy. Other 15 patients did not undergo esophagectomy and received only chemotherapy included DCF therapy. We usually perform esophagectomy about 3 to 4 weeks after the last cycle of DCF therapy. Of all patients, 67 patients (72.8%) underwent 3-field esophagectomy in this study. And also, there were no significant differences in surgical complications between two groups with or without severe neutropenia/ febrile neutropenia (*p* = 0.722) (data were not shown).

The major limitations of our study are the single-center, retrospective design and the small number of patients investigated. However, the current data are based on a prospectively collated database for consecutive patients over a relatively short period. An external validation study involving a sufficient number of patients would be needed to confirm our observations; a multicenter study with a larger number of cases is also warranted.

## Conclusions

The non-use of pegfilgrastim after the first cycle of the DCF regimen to treat esophageal cancer was found to be an independent risk factor for febrile neutropenia, and pegfilgrastim prevented the onset of severe neutropenia and febrile neutropenia. And also, the use of pegfilgrastim can be an option for chemotherapy to achieve the goal of successful completion of scheduled chemotherapy with DCF regimen. Going forward, although the DCF regimen is not yet established as a form of treatment for esophageal cancer, administration of pegfilgrastim as primary prophylaxis during the DCF regimen should be effective.

## Data Availability

Some advocates of clinical data sharing are keen for data to be shared in agreed, standardized formats to facilitate its automated reuse for statistical analysis.

## Abbreviations

5FU: 5-Fluorouracil
cCR: Clinical complete response
CT: Computed tomography
DCF: Docetaxel, cisplatin, and 5-fluorouracil
DSS: Disease-specific survival
NACRT: Neoadjuvant chemoradiotherapy
NACT: Neoadjuvant chemotherapy
OS: Overall survival
pCR: Pathologic complete response
PET: Positron emission tomography
RECIST: Response Evaluation Criteria In Solid Tumors
RFS: Recurrence-free survival
